# The introduction of a minimum wage in Germany and the effects on physical activity participation

**DOI:** 10.1007/s10754-024-09375-2

**Published:** 2024-03-27

**Authors:** Sören Dallmeyer, Christoph Breuer

**Affiliations:** Department of Sport Economics and Sport Management, German Sport University Cologne, Am Sportpark Muengersdorf 6, 50933 Cologne, Germany

**Keywords:** Physical activity, Minimum wage, GSOEP, Public health, I12, I14, I18

## Abstract

The relationship between income and physical activity has been extensively studied. This paper utilizes the introduction of the minimum wage in Germany in 2015 as a quasi-experiment to determine the causal effect of minimum wages on the frequency of physical activity participation. Employing survey data from the German Socio-Economic Panel between 2013 and 2017, regression-adjusted difference-in-difference models combined with matching techniques are estimated. Our findings reveal a notable negative effect immediately after the minimum wage implementation on physical activity frequency. Given that the introduction of the minimum wage did not increase monthly gross income but reduced working hours, it appears that affected individuals exhibit preferences and engage in utility maximization that do not emphasize healthy behaviors. This effect is particularly pronounced among older females in white-collar occupations.

## Introduction

Over the past several decades, numerous countries have implemented minimum wage policies to ensure an adequate standard of living for their citizens. For example, as of now, 22 out of the 27 European Union member states have established some form of statutory minimum wage. Consequently, researchers have extensively investigated the impact of minimum wages on diverse labor market outcomes, such as hourly wages (Dickens & Manning, 2004), total income (Neumark & Wascher, 2007), and employment (Zavodny, 2000). In light of these findings, recent research has begun to explore additional domains potentially influenced by a minimum wage. One emergent strand of literature has examined the effects on physical and mental health. However, the evidence thus far has been inconclusive, with studies reporting positive (Hafner & Lochner, 2022), negative (Horn et al., 2017), and non-significant effects (Maxwell et al., 2022). As a consequence, researchers call for a better understanding of the mechanisms through which minimum wage policies might affect individual health outcomes (Leigh et al., 2019).

One potential mechanism through which minimum wage policies might influence health outcomes is physical activity. The health benefits of physical activity are well-documented (e.g., Warburton et al., 2006), and prior research has demonstrated that socioeconomic disparities at both individual and state levels contribute to inequalities in physical activity participation within populations (Cerin & Leslie, 2008; Pabayo et al., 2018; Petersen et al., 2010). Specifically, individuals with lower incomes, particularly women, have been found to engage in less physical activity (Humphreys & Ruseski, 2015; Kari et al., 2015). Accordingly, with governments worldwide looking for policy interventions to promote health through physical activity participation, understanding the effect of reducing income inequality by implementing a minimum wage may provide valuable insights for policymakers.

The existing literature on this topic is, however, relatively limited and has primarily focused on incremental increases in minimum wages in the United States (Horn et al., 2017; Lenhart, 2019).

This study aims to shed light on this relationship by utilizing the quasi-experimental design of Germany’s introduction of the minimum wage in 2015. Therefore, data from the German Socio-Economic Panel for the years 2013–2017 is used, and a difference-in-difference (DiD) estimator with matching between control and treatment groups is employed to identify the causal effect of the minimum wage on physical activity participation.

## Related literature

Studies examining increases in minimum wages have reported positive effects on self-reported health (Andreyeva & Ukert, 2018), the prevalence of various health conditions (Lenhart, 2017), and mental health (Lebihan, 2023) and mental distress and depression (Bai & Veall, 2023; Kuroki, 2021). Horn et al. (2017) found a significant negative effect on self-reported health but a positive effect on mental health. Maxwell et al. (2022) observed no significant effect. Regarding the specific introduction of a minimum wage, Kronenberg et al. (2017) found no significant effect on mental health in the UK, while Hafner and Lochner (2022) reported a small positive effect on self-rated health in Germany. In their review, Leigh et al. (2019) argued that further research is needed to explore potential pathways between minimum wage and individual health outcomes.

From a theoretical perspective, the relationship between minimum wage and physical activity participation can be described by applying the Grossman model (1972). This economic model on demand for health and healthcare assumes that every individual has a durable capital stock of health which depreciates over time at a certain rate and by investing in either market goods (e.g., health care) or non-market goods (e.g., physical activity), individuals can restore their health. According to the Grossman model, individuals allocate their time between market and non-market activities, aiming to maximize their utility, which is influenced by health status, consumption of goods, and leisure. The model suggests that individuals will invest in health-enhancing behaviors if the marginal benefits, such as improved health, longevity, and higher productivity, outweigh the marginal costs, including time, effort, and resources required for these investments.

An increase in the minimum wage can impact an individual’s investment in health-enhancing behavior through the channels of income and time costs. When health is considered a normal good, an increase in the minimum wage should lead to individuals increasing their health inputs which would improve the individual’s health status. However, it has to be considered that the consumption of unhealthy goods (e.g., alcohol, cigarettes) might increase as well (e.g., Huang et al., 2021). On the other hand, an increase in the hourly wage also increases the opportunity costs of leisure time, potentially leading individuals to substitute physical activity with work or other leisure activities and as a consequence, reduce the time allocated to physical activity. However, potential adverse effects on employment might result in reduced working hours, allowing for more time to engage in the consumption of non-market goods (Burauel et al., 2020).

Existing research has identified both higher income and time costs as important determinants of participation in physical activity. Humphreys and Ruseski (2011) found that a higher income has a significant positive effect on participation in physical activity. This finding is in line with numerous studies which were able to identify this effect consistently (e.g., Downward et al., 2014; Kari et al., 2015). However, regarding the increase in time costs, Humphreys and Ruseski (2011) also showed that higher levels of income had a significant negative relationship with the duration of participation, likely due to higher opportunity costs. In another study, Humphreys and Ruseski (2015) revealed that this two-fold effect is likely depending on the type of physical activity. For example, a higher income is positively associated with participation in swimming or golfing, whereas a negative effect was found for walking or exercising at home.

The relationship between working hours and participation in physical activity is complex, as the time and energy individuals can allocate to leisure activities are influenced by their work schedule and the demands of their job (Kirk & Rhodes, 2011). Numerous studies have found that longer working hours can negatively impact physical activity levels, as individuals may have limited time and energy available for exercise (Fransson et al., 2012). Specifically, employees working extended hours or in jobs with high physical or mental demands may be more likely to experience fatigue and time constraints, limiting their ability to engage in regular physical activity (Kirk & Rhodes, 2011; Schneider & Becker, 2005).

Research examining the impact of minimum wage on physical activity is limited. Horn et al. (2017) explored this effect as part of a supplementary analysis, utilizing a broad measure of physical activity (exercise participation in the last 30 days, yes/no). Their findings suggest that women are more likely to engage in physical activity following a minimum wage increase whereas for men, no effect was found. Lenhart (2019) investigated the association between minimum wage increases and the time individuals allocate to physical activity participation. Employing a DiD approach, the study reveals that a $1 increase in the minimum wage leads to a 20-minute reduction in weekly exercise time. The author posits that this negative relationship may arise from an increase in time dedicated to other leisure pursuits.

This study aims to contribute to this small body of research in several ways. First, as highlighted by Caliendo et al. (2019), the introduction of the minimum wage in Germany presents a compelling case due to the relatively high-income floor compared to other countries, consequently affecting an unusually large proportion of the population. In addition, this case provides an opportunity to explore novel insights into the non-labor market outcomes associated with the introduction of a minimum wage compared to raises of minimum wage levels. Second, by employing a regression-adjusted DiD model with matching, this study follows Leigh et al.‘s (2019) recommendations for addressing the methodological challenge of ensuring comparability between treatment and control groups. Lastly, by utilizing an ordinal measure of participation frequency, this research contributes new evidence, as prior studies have primarily employed binary measures or count measures of duration.

## Research context

After extensive debates and negotiations among policymakers, trade unions, and employer associations, on January 1, 2015, the German government introduced the first statutory uniform minimum wage in the history of the country. The initial minimum wage was set at €8.50 per hour, applicable to all adult employees across the country, regardless of the sector or region. Exempt from the minimum wage policy were minors, interns, and apprentices. Since the implemented floor was relatively high in comparison to other countries, overall, 4.0 million employees, which represent between 10 and 14% of the total workforce, were affected by the regulation (Lesch & Schröder, 2016). Throughout the years, the German government has progressively elevated the minimum wage. In 2017, it was increased to €8.84, followed by subsequent adjustments to €9.19 in 2019, and €9.60 in 2021. On October 1, 2022, the recently elected government enacted the most substantial increase to date, raising the minimum wage to €12.00.

The introduction of the minimum wage in Germany has generated significant interest among researchers, who have examined various aspects of its impact on the labor market and economy. In terms of personal income, the evidence suggests a notable increase in the hourly wage by €0.50 per hour (Burauel et al., 2020). However, Caliendo et al. (2018) demonstrated that this positive effect did not result in increased gross monthly earnings. This can primarily be attributed to significant reductions in contractual hours, as reported by studies from Caliendo et al. (2018) and Pusch and Rehm (2017). Additionally, research has identified no significant impact on poverty risk (Bruckmeier & Bruttel 2021) and only marginal short-term negative employment effects (Caliendo et al., 2018).

## Methods

### Data sources

The analysis of the relationship between the introduction of a minimum wage and physical activity is based on the German Socio-Economic Panel (GSOEP) (GSOEP, 2019). The GSOEP is a German household panel survey conducted annually by the German Institute of Economic Research since 1984. In previous research, the survey data has already been utilized to examine determinants (Breuer & Wicker, 2008) and outcomes of physical activity (Lechner, 2009). With regard to the introduction of a minimum wage, the GSOEP has been used to investigate numerous employment effects (Caliendo et al., 2018) and various well-being outcomes (Gülal & Ayaita, 2020).

The present study focuses on the immediate effect of the implementation of the minimum wage in 2015. Since the information on the participation frequency in physical activity is not available in every wave, it compares the pre-treatment period in 2013 with the post-treatment period in 2015 and 2017.

Given that the focus of the analysis is on the minimum wage, only individuals who are working full-time or part-time are included. Self-employed individuals, interns, and apprentices are not included since the minimum wage does not apply to them. Marginally employed workers were also excluded from the analysis, as comparing their working arrangements to standard contracts presents challenges. Since information on the hourly wage was also available in 2014, observations that showed a change in treatment status compared to 2013 were not considered. The final sample size of the study consists of *n* = 2,258 respondents before the introduction of the minimum wage in 2013 and *n* = 2,448 after the introduction in 2015 and 2017.

### Measures and variables

The outcomes measuring participation frequency in physical activity are assessed as follows: Participation frequency was measured by a four-point scale with the categories at least once a week, at least once a month, less often, or never (*PA frequency*). The four categories are mutually exclusive.^1^ According to the recommendations by the WHO (2010), a certain frequency of participation in physical activity of at least once a week is needed to yield the aimed health benefits. Hence, a dummy variable for the category of participation in physical activity at least once a week (*PA weekly*) was used as a binary dependent variable, with 1 indicating weekly participation.

To determine an accurate hourly wage, the monthly gross income—excluding bonus payments—is divided by the number of working hours. Since working hours were provided on a weekly basis, they were multiplied by 4.33. This number is used because there are, on average, 4.33 weeks in a month (accounting for both 30 and 31-day months). The approach is consistent with the recommendation by Dütsch et al. (2019). The *treatment* status variable indicates whether someone was affected by the minimum wage (= 1) or not (= 0). The treatment group comprises individuals who earned an hourly wage below €8.50 prior to the introduction of the new minimum wage in 2013. The control group consists of individuals who earned an hourly wage greater than €8.50 but no more than €12.75 (50% higher). This upper limit enables the formation of socio-demographically similar control and treatment groups. This approach has been previously employed in similar contexts by Gülal and Ayaita (2020) and Reeves et al. (2017). Additionally, the analysis includes two time-specific dummy variables for the years 2015 and 2017 to identify the post-minimum wage periods. These variables are assigned a value of 1 for the corresponding post-minimum wage years and 0 for the baseline year of 2013.

Furthermore, the dataset offers comprehensive information on socio-demographic and job-related characteristics (Table 1). The following socio-demographic control variables that influence the frequency of physical activity are included: *Age* and *Age*^*2*^ to account for a non-linear relationship, a binary indicator for health problems (*Bad health*), presence of children (*Children*), five distinct dummy variables representing various educational levels (*No degree*, *Main school*, *Secondary school*, *Field-specific*, and *A-level*), an indicator for a university degree (*University degree*), household size (*HH size*), gender (*Male*), and marital status (*Married)*.

**Table 1:** Overview of conditional variables

Variables	Description	Scale
*Dependent variables*		
PA frequency	Participation frequency in physical activity (4-point scale)	Ordinal
PA weekly	Participation in physical activity at least once a week (1 = yes)	Binary
*Socio-demographic*		
Age	Age (in years)	Metric
Age^2^	Age squared	Metric
Bad health	Self-reported health status (0 = other; 1 = bad health)	Binary
Children	Presence of a child (1 = yes)	Binary
Education		
No degree	No degree (yes = 1)	Binary
Main school	Main school degree (yes = 1)	Binary
Secondary school	Secondary school degree (yes = 1)	Binary
Field-specific	Field-specific degree (yes = 1)	Binary
A-level	A-level (yes = 1)	Binary
University degree	University degree (yes = 1)	Binary
HH size	Number of persons living in the household	Metric
Male	Gender (0 = female; 1 = male)	Binary
Married	Marital status (0 = other; 1 = married)	Binary
*Job-related*		
Tenure	Tenure (in years)	Metric
Company size		
< 10	Number of employees < 10 (yes = 1)	Binary
10–100	Number of employees 10–100 (yes = 1)	Binary
101–2000	Number of employees 101–2000 (yes = 1)	Binary
> 2000	Number of employees > 2000 (yes = 1)	Binary
Education fit	Fit between education and match (yes = 1)	Binary
Job change	Job change in the past (yes = 1)	Binary
Job status		
Blue-collar	Blue-collar job (yes = 1)	Binary
White-collar	White-collar job (yes = 1)	Binary
Part-time	Part-time job (yes = 1)	Binary
Temporal job	Temporal job (yes = 1)	Binary

For job-related controls, the study incorporates the tenure with the employer (*Tenure*), the size of the employer based on the number of employees (*< 10; 10–100; 101–2000; >2000*), the compatibility of the job with the individual’s education (*Education fit*), whether there was a job change in the previous year (*Job change*), and whether the job is part-time (*Part-time*) or temporary (*Temporal job*). Information on the type of job (*Blue-collar* and *White-collar*) and based on the NACE branch codes, seventeen dummy variables controlling for the industry are considered. In addition, every model includes state fixed effects to control for regional differences.

### Empirical analysis

The study applies a DiD approach to estimate the causal effect of the introduction of the minimum wage in 2015 on physical activity in Germany. The DiD captures how participation in physical activity in the treatment group changes in comparison to the control group. The following notation gives the econometric model:

1 $$\begin{aligned} {Y}_{it} & = {\beta }_{0}+{\beta }_{1}{Treatment}_{i}+ {\beta }_{2}{Post2015}_{t}+{\beta }_{3}{Post2017}_{t}+{\beta }_{4}{Treatment}_{i} \\ & \quad *Post{2015}_{t} +{\beta }_{5}{Treatment}_{i}*Post{2017}_{t}+{\beta }_{6}{X}_{it}+{\beta }_{7}{Z}_{it}+{\theta }_{i}+{\varepsilon }_{it}\end{aligned}$$

${Y}_{it}$ describes the physical activity outcome variable varying by individual *i* and by time *t*. The treatment effect on the treatment group (ATT) is identified by the interactions between the treatment group indicator variable ${Treatment}_{i}$and the variables reflecting the post-minimum wage years ${Post2015}_{t}$ and ${Post2017}_{t}$. To improve the precision of the model the vector ${\beta }_{4}{X}_{it}$ captures the effect of the time-variant socio-demographic control variables and ${\beta }_{5}{Z}_{it}$ the effect of job-related control variables. Individual fixed effects are included with ${\theta }_{i}$.

To ensure comparability between the control and treatment groups based on their observable characteristics, a regression-adjusted DiD matching strategy was employed, following the method proposed by Heckman et al. (1997). In the first step, a probit model estimates propensity scores, representing the probability of being affected by the minimum wage, conditional on the observable characteristics. These include the aforementioned socio-demographic and job-related control variables. Next, kernel matching with the epanechnikov kernel function and with bandwidth of 0.06 was conducted. This method assigns weights to individuals based on their propensity scores and has been widely adopted in previous studies (Hafner & Lochner, 2022; Marcus, 2014). The results were robust for different specifications of the matching procedure such as a logit model in the first stage, different kernel functions, and bandwidths. The results are available upon request.

When analyzing both outcomes—frequency of physical activity (PA) and weekly PA participation—we employed individual fixed effects models. Lechner et al. (2016) argue that incorporating individual fixed effects enhances the precision of DiD models, particularly when time-varying panel non-response might affect the assumption of parallel trends. Given the fluctuation in our sample size across the time periods, the adoption of individual fixed effects models is posited to yield more consistent estimations. Both outcomes yielded similar results in (ordered) logit models for the non-matching scenarios. All models are estimated with robust standard errors.

To identify the causal effect of the minimum wage on the frequency of physical activity participation, the assumption is made that the difference in the change in physical activity behavior between the treatment and control groups is only attributable to the introduction of the minimum wage. Consequently, the physical activity outcomes for both groups would have exhibited similar changes over time had the minimum wage not been implemented (common trend assumption). The inclusion of numerous time-variant socio-demographic, job-related control variables and individual fixed effects should increase the likelihood of the common trend assumption holding true. Additionally, an event study model spanning the years 2009–2017 is estimated. In an event study, lag and lead variables related to the event of interest (i.e., the introduction of the minimum wage) are incorporated, allowing for the examination of any significant pre-trends in terms of differences in physical activity participation between the control and treatment groups. In our model, the years 2009 and 2011 are included as pre-trend years.

## Results

### Descriptive statistics

Table 2 displays the mean values of the control and treatment group characteristics before and after matching. In general, the results suggest that females with lower education levels and those working for smaller employers are more likely to be affected by the minimum wage. Prior to matching, the descriptive statistics reveal only a few significant differences for socio-demographic characteristics (e.g., *Male*, *Married*) but substantial disparities in job-related characteristics, with most job-related controls showing significant differences. Following the matching process, nearly all significant differences disappear, indicating a suitable comparison between the treatment and control groups.

**Table 2:** Summary statistics of treatment and control group in 2013 (mean values)

	Treated	Controls	
		Unmatched	Matched
*Socio-demographic*			
Age	41.874	41.295	42.151
Age^2^	1,837.299	1,903.628	1,895.194
Bad health	0.152	0.125	0.154
Children	0.535	0.541	0.542
Education			
No degree	0.113	0.101	0.095
Main school	0.261	0.256	0.260
Secondary school	0.507	0.481	0.510
Field-specific	0.022	0.041**	0.022
A-level	0.093	0.119	0.114
University degree	0.071	0.096**	0.080
HH size	2.929	3.060	2.932
Male	0.310	0.417***	0.294
Married	0.437	0.564***	0.434
*Job-related*			
Company tenure	5.685	7.444***	5.883
Company size			
< 10	0.331	0.198***	0.325
10–100	0.351	0.353	0.341
101–2000	0.214	0.307***	0.220
> 2000	0.104	0.142***	0.114
Education fit	0.379	0.514***	0.375
Job change	0.266	0.179***	0.249
Job status			
Blue-collar	0.381	0.355	0.370
White-collar	0.619	0.645	0.630
Part-time	0.504	0.383***	0.503
Temporal job	0.212	0.160***	0.200

Values refer to the pre-treatment period in 2013. *** *p* < 0.01, ** *p* < 0.05, * *p* < 0.1 indicate p-values from t-test testing for significant differences between the treatment and either matched or unmatched control groups.

### Main results

Table 3 presents the impact of the minimum wage introduction in 2015 on the frequency of physical activity participation. Columns 1–3 display the estimates for participation frequency measured on a 4-point scale, while columns 4–6 depict the results for the binary outcome of participating at least once a week. Columns 1 and 4 show the results without matching including the socio-demographic and job-related control variables. Columns 2 and 5 describe the estimates with fixed effects and Columns 3 and 6 showcase the regression-adjusted DiD results with matching. A significant negative effect is observed for both frequency outcomes across all models in the first year after the introduction of the minimum wage. For 2017, the DiD coefficient becomes insignificant in all models. The effects are robust when including individual fixed effects. The inclusion of matching weights leads to considerably larger effects. The impacts of the considered socio-demographic control variables, which are not shown, align with previous research, illustrating, for instance, a positive relationship between education and physical activity, as well as a negative relationship with health problems.

**Table 3:** DiD-analysis of the introduction of minimum wage and physical activity (2013–2017)

	PA frequency			PA weekly		
	(1)	(2)	(3)	(4)	(5)	(6)
DiD (2015)	-0.191**	-0.186**	-0.228**	-0.078**	-0.085**	-0.117***
	(0.095)	(0.094)	(0.105)	(0.035)	(0.037)	(0.040)
DiD (2017)	-0.135	-0.042	-0.016	-0.052	-0.039	-0.031
	(0.106)	(0.110)	(0.119)	(0.039)	(0.042)	(0.046)
Observations	4,706	4,706	4,587	4,706	4,706	4,587
Control variables	Yes	Yes	Yes	Yes	Yes	Yes
Indiv. fixed effects		Yes	Yes		Yes	Yes
Matching			Yes			Yes

All models include state and industry fixed effects. Clustered standard errors in parentheses; *** *p* < 0.01, ** *p* < 0.05, * *p* < 0.1.

### First-order effects

Consistent with the Grossman model, we observed that the hourly wage increased by €0.486 between the control and treatment groups in 2015 and by €1.092 in 2017 (Table 4). However, there was no significant variation in the monthly gross income across both groups in 2015. In 2017, the gross income increased by €72.590. Simultaneously, contractual hours were reduced by 0.840 h in 2015 and 1.277 h by 2017 which could indicate that to accommodate the rise in hourly wages, either employers or employees reduced working hours. However, actual working hours reported by the employees were not significantly affected.

**Table 4:** DiD-analysis of the introduction of minimum wage and income and working hours (2013–2017)

	(1)	(2)	(3)	(4)
	Hourly wage	Gross income	Contr. work hours	Act. work hours
DiD (2015)	0.486***	34.422	-0.840**	-0.677
	(0.192)	(25.934)	(0.383)	(0.470)
DiD (2017)	1.092***	72.590**	-1.277**	-0.859
	(0.248)	(32.298)	(0.525)	(0.525)
Observations	4,636	4,636	4,636	4,710
Control variables	Yes	Yes	Yes	Yes
Indiv. fixed effects	Yes	Yes	Yes	Yes
Matching	Yes	Yes	Yes	Yes

All models are regression-adjusted DiD models with matching weights. State and industry fixed effects are included. Robust standard errors in parentheses; *** *p* < 0.01, ** *p* < 0.05, * *p* < 0.1.

### Heterogeneity of effects

Tables 5 and 6 present the DiD effects across various subsamples. The first panel A shows the impact on male and female participants. The findings demonstrate a substantial negative effect on women’s physical activity participation frequency after the introduction of the minimum wage in 2015, while no significant effect is observed for men. Regarding, the first-order effects, the increases in hourly wage and the reduction in working hours can only be observed for females. Panel B distinguishes between blue-collar and white-collar job holders. Notably, a significant negative effect on physical activity participation frequency is observed for individuals in white-collar jobs, whereas no significant effects are found for those in blue-collar occupations. The hourly wage increased only for people in white-collar occupations, the working hours, however, decreased only for blue-collar jobs. Panel C examines three age groups, revealing significant negative effects for the oldest age groups 18–36 and 36–49 whereas the oldest age groups mostly exhibit no significant impact on physical activity frequency. The oldest age group is also the only group where a decrease in contractual and actual working hours is found. Lastly, Panel D differentiates between levels of education. Individuals below a secondary school degree (less than 10 years in school) are categorized as *low education* while all others are grouped as *high education*. The findings indicate that the introduction of the minimum wage has a significantly negative effect on physical activity participation frequency for individuals with lower and higher educational attainment, while a significant negative effect on participation duration can only be observed for individuals with lower education. Interestingly, the positive effect on hourly wage only occurs for individuals with higher education whereas the reduction in working hours is only found for the group of individuals with lower education.

**Table 5:** DiD-subsample-analysis of the introduction of the minimum wage and physical activity (2013–2017)

	PA frequency		PA weekly	
	DiD (2015)	DiD (2017)	DiD (2015)	DiD (2017)
*Panel A: Gender*				
Male	-0.133	-0.116	-0.005	-0.059
	(0.151)	(0.212)	(0.061)	(0.100)
Female	-0.313**	-0.010	-0.173***	-0.051
	(0.134)	(0.145)	(0.050)	(0.054)
*Panel B: Job status*				
Blue-collar	-0.119	-0.206	-0.073	-0.108
	(0.174)	(0.204)	(0.674)	(0.082)
White-collar	-0.410***	-0.049	-0.182***	-0.021
	(0.157)	(0.157)	(0.060)	(0.063)
*Panel C: Age*				
18–35	-0.455*	0.088	-0.154	-0.009
	(0.263)	(0.291)	(0.103)	(0.128)
36–49	-0.335*	-0.216	-0.335*	-0.216
	(0.181)	(0.190)	(0.181)	(0.190)
50+	0.093	-0.111	0.009	-0.024
	(0.244)	(0.288)	(0.088)	(0.100)
*Panel D: Education*				
Higher education	-0.253	0.032	-0.097*	-0.031
	(0.181)	(0.242)	(0.052)	(0.055)
Lower education	-0.184	0.011	-0.139**	-0.023
	(0.137)	(0.138)	(0.068)	(0.089)
Control variables	Yes	Yes	Yes	Yes
Indiv. fixed effects	Yes	Yes	Yes	Yes
Matching	Yes	Yes	Yes	Yes

All models are regression-adjusted DiD models with matching weights. Lower education refers to individuals with a degree below secondary school. State and industry fixed effects are included. Robust standard errors in parentheses; *** *p* < 0.01, ** *p* < 0.05, * *p* < 0.1.

**Table 6:** DiD-subsample-analysis of the introduction of the minimum wage and first order effects (2013–2017)

	Hourly wage		Gross income		Contr. work hours			Act. work hours	
	DiD (2015)	DiD (2017)	DiD (2015)	DiD (2017)	DiD (2015)	DiD (2017)		DiD (2015)	DiD (2017)
*Panel A: Gender*									
Male	-0.044	0.108	40.166	-44.215	1.441	-0.728		0.052	-2.109*
	(0.296)	(0.451)	(56.539)	(69.260)	(0.946)	(0.829)		(1.072)	(1.148)
Female	0.483**	1.109***	12.368	46.751	-1.227***	-1.617**		-1.057*	-1.238*
	(0.203)	(0.318)	(27.868)	(44.373)	(0.441)	(0.673)		(0.579)	(0.686)
*Panel B: Job status*									
Blue-collar	0.350	0.429	27.796	-7.536	-0.684	-1.549**		-1.075	-1.771**
	(0.213)	(0.298)	(40.980)	(47.095)	(0.652)	(0.739)		(0.880)	(0.870)
White-collar	0.572**	1.295***	47.504	98.154**	-0.393	-0.750		-0.076	-0.209
	(0.236)	(0.354)	(33.419)	(44.062)	(0.491)	(0.771)		(0.649)	(0.741)
*Panel C: Age*									
18–35	0.540	0.870	82.535	118.452	0.006	-0.031		0.834	1.147
	(0.423)	(0.620)	(66.586)	(91.612)	(0.948)	(1.176)		(1.191)	(1.635)
36–49	0.656**	0.701**	58.307	17.492	-0.557	-0.942		-0.158	-0.693
	(0.277)	(0.357)	(36.347)	(40.781)	(0.551)	(0.726)		(0.752)	(0.891)
50+	-1.382	0.989	-149.103*	-5.431	-1.308**	-1.701**		-1.588*	-1.256
	(0.942)	(0.985)	(78.804)	(104.696)	(0.536)	(0.691)		(0.903)	(0.805)
*Panel D: Education*									
Higher education	0.682***	1.179***	87.619**	99.661**	-0.142	-0.836		0.133	-0.592
	(0.249)	(0.324)	(39.602)	(47.405)	(0.471)	(0.687)		(0.555)	(0.633)
Lower education	-0.140	0.427	-80.783*	-54.692	-1.568**	-1.900**		-2.535**	-1.449
	(0.371)	(0.488)	(47.683)	(68.310)	(0.738)	(0.811)	(1.078)		(1.171)
Control variables	Yes	Yes	Yes	Yes	Yes	Yes	Yes		Yes
Indiv. fixed effects	Yes	Yes	Yes	Yes	Yes	Yes	Yes		Yes
Matching	Yes	Yes	Yes	Yes	Yes	Yes	Yes		Yes

All models are regression-adjusted DiD models with matching weights. Lower education refers to individuals with a degree below secondary school. State and industry fixed effects are included. Robust standard errors in parentheses; *** *p* < 0.01, ** *p* < 0.05, * *p* < 0.1

### Common trend assumption and robustness checks

**Fig. 1:**
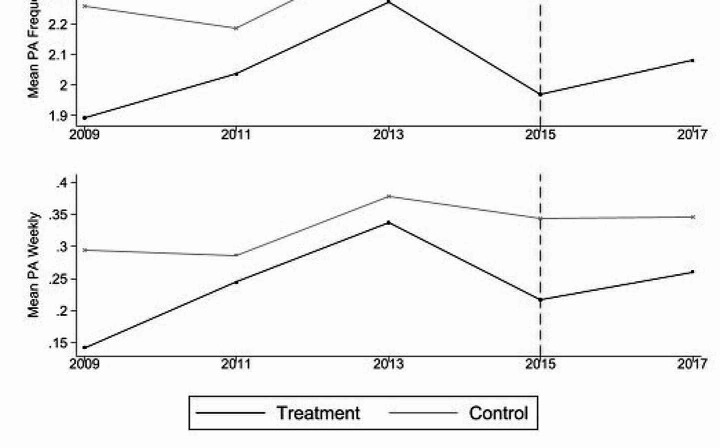
Development of average physical activity (PA Frequency and PA Weekly) for treatment and control group (2009–2017)

**Fig. 2:**
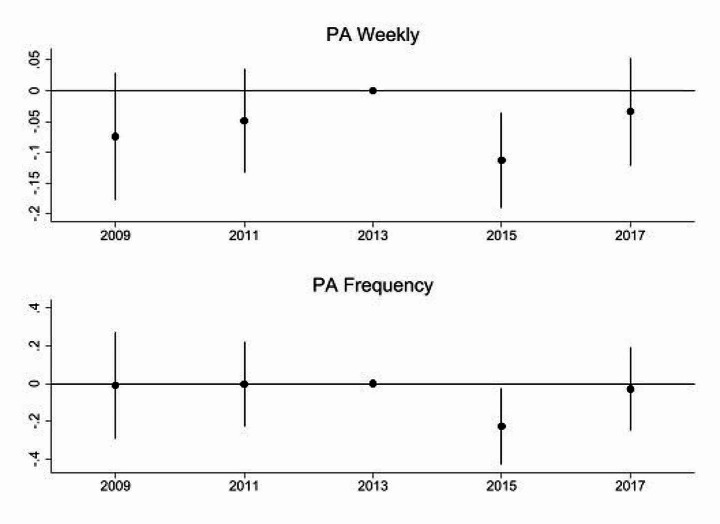
Event-study to test common trend assumption. All models are regression-adjusted DiD models with matching weights. State and industry fixed effects are included (2009–2017)

Figure 1 presents the development of average physical activity levels across both the treatment and control groups. The identifying assumption is that both groups would have developed similarly if the minimum had not been introduced. Figure 2 displays the results of the event study conducted to address this common trend assumption. For both physical activity frequency outcomes, the interaction effects between the years 2009 and 2011 and the treatment variable MW are negative but non-significant, as the p-values of all coefficients are higher than 0.1. This finding suggests that the common trend assumption is not violated. However, given the reduced sample size in the pre-treatment period, interpreting the results requires caution. This is in line with earlier studies, which suggest that the limited power of pre-trend analyses might lead to insignificant findings (Roth, 2022).

To ensure that our chosen threshold of an hourly wage of €12.75 (150%) defining the control group is not driving the results, the regression-adjusted DiD models with matching were estimated for thresholds of €10.00 (115%) and €17.00 (200%) (Table 7). Furthermore, the same models were estimated without individuals earning an hourly wage between €8.50 and €10.00, as previous research has demonstrated that potential spillover effects on individuals earning just above the minimum wage of €8.50 could bias the results (Aretz et al., 2013). Across all specifications, the results remain consistent and do not vary considerably depending on the choice of threshold.

**Table 7:** Robustness check for different hourly wage thresholds for the control group

	(1)	(2)
	PA frequency	PA weekly
*Panel E: Hourly wages < €10.00*		
DiD (2015)	-0.265**	-0.151***
	(0.123)	(0.048)
DiD (2017)	-0.008	-0.056
	(0.130)	(0.049)
*Panel F: Hourly wages > €10.00 and < €17.00*		
DiD (2015)	-0.196*	-0.083**
	(0.109)	(0.042)
DiD (2017)	-0.025	-0.016
	(0.128)	(0.051)
*Panel G: Hourly Wages < €17.00*		
DiD (2015)	-0.229**	-0.109***
	(0.100)	(0.039)
DiD (2017)	-0.012	-0.030
	(0.118)	(0.046)
Control variables	Yes	Yes
Indiv. fixed effects	Yes	Yes
Matching	Yes	Yes

All models are regression-adjusted DiD models with matching weights. State and industry fixed effects are included. Robust standard errors in parentheses; *** *p* < 0.01, ** *p* < 0.05, * *p* < 0.1.

Considering the potential endogeneity of the individual health status, the models were estimated excluding self-reported health problems as a control variable. The robustness checks confirmed that the exclusion had only a marginal impact on the results, indicating that the findings remained consistent despite the omission (Table 8).

In order to investigate whether individuals have allocated the additional time gained from fewer working hours and decreased physical activity to alternative leisure activities, Table 9 presents the impact of the minimum wage introduction on engagement in high culture (like visiting operas or museums), low culture (such as cinema or concerts), voluntary work, and active arts. The findings predominantly show no significant effects, except for a modest significant increase in the frequency of low cultural activities in 2017 (Table 9).

### Discussion and conclusion

The study takes advantage of the introduction of a minimum wage in Germany as a quasi-experiment to investigate the relationship between minimum wage policy and physical activity participation frequency. Respondents are categorized based on their hourly wage, resulting in a treatment group affected by the policy and a control group that remains unaffected. The random assignment of individuals to either group allows for the use of a regression-adjusted DiD model to estimate the causal effect. In line with Leigh et al.’s (2019) recommendations, descriptive statistics emphasize the importance of employing a matching procedure to address significant differences between the control and treatment groups.

**Fig. 3:**
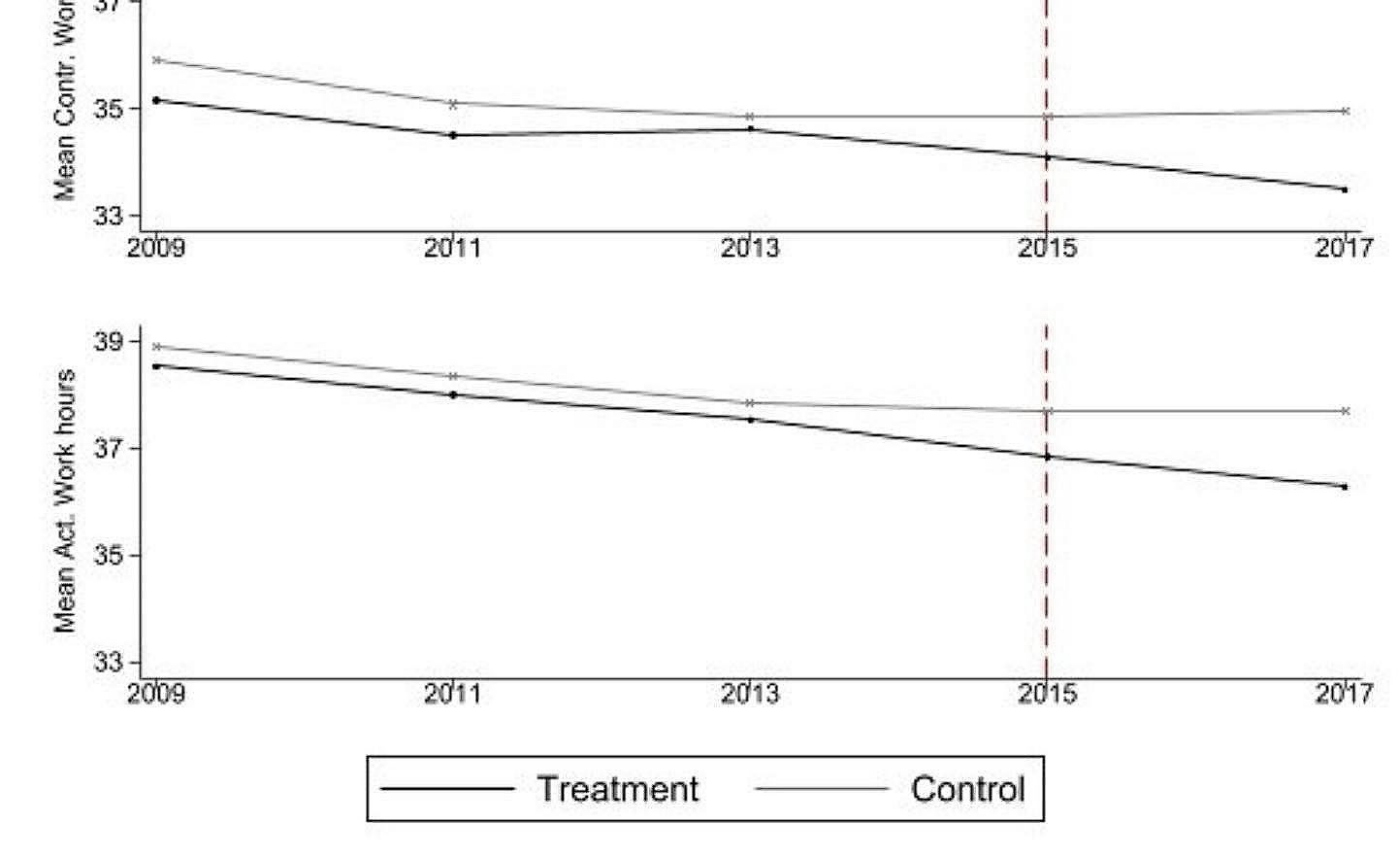
Development of average working hours (Contractual and Actual) for treatment and control group (2009–2017)

The findings demonstrate an immediate significant negative effect of the minimum wage implementation on physical activity participation frequency and the probability of meeting the World Health Organization’s (2010) recommended weekly frequency threshold of at least once a week. These results align with Lenhart (2019), who found a significant negative relationship between minimum wage increases and physical activity duration. This could potentially help to explain an underlying mechanism behind the negative health consequences of minimum wage policies observed in some studies (e.g., Buszkiewicz et al., 2021; Horn et al., 2017). A closer examination of the first-order effects reveals that the minimum wage introduction did not result in higher total income, as increased hourly wages were counterbalanced by a significant reduction in working hours (Fig. 3). Similar effects have been documented in previous research studying Germany’s minimum wage. Studies have demonstrated that both employers and employees adopt this approach as a method of adjustment. For instance, in a survey conducted by Bellmann et al. (2016), employers identified a reduction in contractual working hours as the primary strategy for adapting to the minimum wage. On the other hand, research has shown that in particular marginally employed individuals have an incentive to reduce their working hours to avoid exceeding income or allowance thresholds or to avoid shifting into employment subject to social security contributions. For example, Caliendo et al. (2018) demonstrate using a difference-in-differences approach with SOEP data on the regional depth of the minimum wage impact that the reduction in contractual working hours is particularly pronounced among part-time employees and mini-jobbers.

Drawing upon Grossman’s (1972) model, it can be inferred that the change in opportunity costs led to a re-evaluation of the marginal costs and marginal benefits for optimal time and resource allocation to maximize utility. On average, individuals in the treatment group did not prioritize health-promoting behaviors, opting instead to allocate additional time to other leisure activities (Lenhart, 2019) or engage in behaviors detrimental to physical activity participation, such as unhealthy eating (Andreyeva & Ukert, 2018), alcohol consumption (e.g., Adams et al., 2012), or smoking (Huang et al., 2021).

When examining the heterogeneity of effects, the results indicate that the negative impact is particularly evident for women with lower education working in white-collar jobs from age 18–50. This could potentially suggest that individuals from these subgroups seem to prefer non-healthy activities over participating in physical activity. For instance, Mullahy and Robert (2010) have demonstrated that individuals with lower educational attainment are less likely to allocate time to health-producing leisure activities such as physical activity. The significant negative effect for white-collar jobs, compared to blue-collar jobs, could imply that the reduction in working time for blue-collar jobs might have led to lower physical demands, thereby freeing up resources for physical activity participation during leisure time (Kirk & Rhodes, 2011). Concerning the gender-specific effects, another mechanism driving the result could be the unequal distribution of care work between men and women. According to the OECD (2017), Germany ranks relatively high in terms of the share of house and childcare work women perform compared to their male partners, and Morrissey (2023) has already demonstrated that increases in minimum wages lead to mothers spending more time with their children, while the effects for men were non-significant.

Based on these findings, the introduction of the minimum wage in Germany cannot be deemed an appropriate policy to increase physical activity participation levels among individuals with lower socio-economic status. Instead, potential negative effects on physical activity participation, leading to adverse health impacts, should be included in public discussions about the costs and benefits of such labor market regulations, particularly with previous studies already indicating negative health effects of minimum wages. However, it is essential to bear in mind that if minimum wages result in higher monthly income, a subsequent income effect could alter the outcome and potentially offset the negative substitution effects. Additionally, policymakers and employers should recognize that some sub-groups are especially affected and, consequently, provide public health education and opportunities for physical activity alongside the introduction of the minimum wage.

This study has several limitations that future research should address. First of all, only broad measures of physical activity frequency were used without information on intensity, making it difficult to draw conclusions about specific activities. Considering the significant role of opportunity costs in this context, a detailed examination of the time intensity of different activities could be valuable in this regard (Humphreys & Ruseski, 2011). Second, a better understanding of the persistence of the effects is needed. The results indicate that a significant positive effect occurs only in the year immediately following the introduction of the minimum wage. This might be attributable to the smaller sample size in 2017, but it could also potentially suggest adjustments by employers and employees in terms of first-order effects and physical activity behavior. Lastly, future research should attempt to illuminate the mechanisms responsible for the negative effects on physical activity participation. Given that our analysis reveals no significant impact on other leisure activities and lacks data on behaviors such as smoking or alcohol consumption, further research is needed to understand how individuals have repurposed the time freed from reduced work hours and physical activity.

**Table 8:** Robustness check for DiD-analysis without self-reported health problems (Bad health) as a control variable

	PA frequency			PA weekly		
	(1)	(2)	(3)	(4)	(5)	(6)
DiD (2015)	-0.190**	-0.187**	-0.224**	-0.078**	-0.085**	-0.122***
	(0.095)	(0.094)	(0.105)	(0.035)	(0.037)	(0.040)
DiD (2017)	-0.136	-0.044	-0.024	-0.053	-0.039	-0.040
	(0.106)	(0.110)	(0.119)	(0.039)	(0.042)	(0.046)
Observations	4,710	4,710	4,594	4,710	4,710	4,594
Control variables	Yes	Yes	Yes	Yes	Yes	Yes
Indiv. fixed effects		Yes	Yes		Yes	Yes
Matching			Yes			Yes

All models include state and industry fixed effects. Clustered standard errors in parentheses; *** *p* < 0.01, ** *p* < 0.05, * *p* < 0.1.

**Table 9:** DiD-analysis of the introduction of minimum wage and different leisure activities (2013–2017)

		(1)	(2)	(3)	(4)	(5)	(6)	(7)	(8)
		High cult	High cult week	Low cult	Low cult week	Vol work	Vol work week	Active arts	Active arts week
DiD (2015)		0.027	0.016	0.076	-0.007	-0.015	-0.004	0.122	-0.011
		(0.062)	(0.011)	(0.059)	(0.009)	(0.071)	(0.022)	(0.081)	(0.027)
DiD (2017)		0.013	0.005	0.031	0.029*	-0.071	0.002	0.111	0.019
		(0.060)	(0.007)	(0.063)	(0.016)	(0.076)	(0.023)	(0.087)	(0.028)
Observations		4,595	4,595	4,591	4,591	4,582	4,582	4,590	4,590
Control variables		Yes	Yes	Yes	Yes	Yes	Yes	Yes	Yes
Indiv. fixed effects		Yes	Yes	Yes	Yes	Yes	Yes	Yes	Yes
Matching		Yes	Yes	Yes	Yes	Yes	Yes	Yes	Yes

All models include state and industry fixed effects. Clustered standard errors in parentheses; *** *p* < 0.01, ** *p* < 0.05, * *p* < 0.1.

## Footnotes

1 In 2013, the variable was assessed using a 5-point scale that included a “daily” option. To align with the typical 4-point scale, we combined the “daily” and “at least once a week” responses.”
